# Short-term group schema therapy for mixed personality disorders: a pilot study

**DOI:** 10.3389/fpsyg.2014.01592

**Published:** 2015-01-22

**Authors:** Sally A. Skewes, Rachel A. Samson, Susan G. Simpson, Michiel van Vreeswijk

**Affiliations:** ^1^School of Psychology, Social Work and Social Policy, University of South AustraliaAdelaide, SA, Australia; ^2^G-kracht Psychomedisch Centrum BVDelft, Netherlands

**Keywords:** personality disorders, schema therapy, group, case series, comorbidity

## Abstract

Schema Therapy has shown promising results for personality disorders but there is a limited evidence base for group schema therapy (ST-g) with mixed personality disorders. The aim of this study was to explore the feasibility, acceptability, and preliminary effectiveness of ST-g in a sample of eight participants with mixed personality disorders (with a predominant diagnosis of avoidant personality disorder) and high levels of comorbidity. Treatment was comprised of 20 sessions which included cognitive, behavioral, and experiential techniques. Specific schema-based strategies were chosen for a diagnostically mixed group of personality disorder clients. Six participants attended until end of treatment and two dropped-out before mid-treatment. All outcome measures showed changes with large effect sizes in avoidant personality disorder symptom severity, depression and anxiety levels between pre-therapy and follow-up. Four participants achieved a loss of personality disorder diagnosis at the end of therapy. By follow-up, five participants had achieved a loss of diagnosis, suggesting that participants derived ongoing benefits from the group even after treatment ended. Six participants no longer met criteria for depression at the end of treatment and this was maintained for all participants at 6-month follow-up. At follow-up, the majority of participants showed clinically significant change on the Global Symptom Index (GSI). For the Schema Mode Inventory (SMI) maladaptive modes, the majority of participants showed improvement at follow-up. At follow-up, 40% of participants showed clinically significant change on the SMI adaptive modes. Qualitative feedback indicates that the group helps to normalize participants' psychological experiences and difficulties and promotes self-expression and self-disclosure, while reducing inhibition. Preliminary results suggest that short-term ST-g may benefit those with mixed personality disorders, but generalizability is limited by the small sample size and lack of control group.

## Introduction

Personality disorders are highly prevalent in clinical settings. Approximately one-third of clients in outpatient clinical settings are diagnosed with a personality disorder (Zimmerman et al., 2005). The majority of individuals diagnosed with a personality disorder meet criteria for more than one. Avoidant, borderline, and obsessive-compulsive personality disorders are among the most frequently specified diagnoses (Zimmerman et al., 2005). Personality disorders are notoriously difficult to treat and often require long-term treatment with psychotherapy (National Institute of Clinical Excellence, 2009). One psychotherapy that shows promise for effectively treating a range of personality disorders is Schema Therapy.

Schema Therapy (ST) is one of the third wave innovative therapies that have developed specifically for treating personality disorders and other complex, chronic clinical presentations. ST has proven to be an efficacious and cost effective treatment for Borderline Personality Disorder (BPD) (Giesen-Bloo et al., 2006; van Asselt et al., 2008; Farrell et al., 2009; Nadort et al., 2009; Masley et al., 2011). A multicenter randomized controlled trial (RCT; Giesen-Bloo et al., 2006) with 86 patients compared ST to Transference-Focused Psychotherapy (TFP) over a 3 year period (with a follow-up 1 year later). ST was more effective than TFP both in terms of recovery from BPD and all outcome measures, and had a lower rate of dropouts over 3 years (27% vs. 50%). ST also proved to be more cost effective than TFP (less costs and better effects) (van Asselt et al., 2008). Another study, Nadort et al. (2009) found ST to be superior to treatment as usual (TAU) for BPD. In light of the promising results for ST with BPD, the application of ST is currently being extended to other personality disorders, treatment settings, and client populations. A recent RCT found ST was more effective than clarification-oriented psychotherapy for cluster C personality disorders (Bamelis et al., 2014). Another RCT, Ball et al. (2011) compared dual-focus schema therapy with individual drug counseling as adjuncts to treatment as usual in a population of 105 substance dependent patients with personality disorders. The study found that there was a comparable symptom reduction in both conditions; however, drop-out rates were high across conditions and individual drug counseling resulted in more sustained reductions than did dual-focus schema therapy in several symptoms for several personality disorders. Aside from this, only a handful of preliminary studies have investigated the effectiveness of ST in the treatment of personality disorders other than BPD, with very few in group settings (e.g., Simpson et al., 2010; van Vreeswijk et al., 2012; Renner et al., 2013; Videler et al., 2014).

The emergence of group therapy protocols has been an important development in the growth of ST. It is thought that group therapy provides important curative factors, including corrective emotional learning experiences, a unique opportunity for clients to practice new behavioral and coping skills in a de-shaming environment, and opportunities for vicarious learning (Farrell et al., 2009). In a study which compared a group schema therapy approach (Group ST) to TAU, Farrell and Shaw found Group ST demonstrated significantly larger treatment effects, fewer dropouts, shorter duration, and required less therapist time (Farrell et al., 2009). Similar promising results were found in a pilot study (Dickhaut and Arntz, 2013) assessing the effectiveness of a combination of individual and group schema therapy for BPD patients provided over a 2 year period. A shorter group schema cognitive behavioral therapy (SCBT-g) approach has been developed by van Vreeswijk and Broersen (2006, 2013) and successfully piloted in eating disorder patients (Simpson et al., 2010). In a more recent study of 63 patients, SCBT-g was associated with changes on all outcome measurements with moderate to high effect sizes, with 53.2% of the patients showing a significant reduction in severity of psychiatric symptoms and schemas and modes. Compared to the Group ST model of Farrell and Shaw (1994, 2012), Farrell et al. (2014), the SCBT-g therapy is more structured and focuses more on the original schema model whereas the Group ST model of Farrell and Shaw focuses more on schema modes. An international multicenter RCT is currently underway investigating Group ST for BPD using the protocol developed by Farrell and Shaw (2012). Moreover, a multicenter trial comparing group schema therapy to traditional group CBT for patients with generalized social phobia and comorbid avoidant personality has commenced (Greeven et al., 2013).

Preliminary evidence supports the use of ST in group treatment for BPD; however, evidence supporting the use of group schema therapy with patients with other personality disorders is sparse. This paper described a pilot study using short-term group schema therapy (ST-g) in a case series of eight patients with Cluster A, B, and C personality disorders and high levels of comorbidity.

## Design

The present study utilized a single group pre- and post-pilot study in order to investigate the outcome of ST-g for a group of participants with mixed personality disorders in an outpatient university clinic. The aim of this study was to explore the feasibility, acceptability, and preliminary effectiveness of ST-g. This pilot study is a requisite initial step in informing the feasibility of a larger scale study investigating the efficacy of ST-g in patients with mixed personality disorders.

## Method

### Participants

Informed consent was obtained from all patients and ethics approval was granted. Patients were referred to the group from the University of South Australia psychology clinic, private psychologists, and non-government organizations. Patients were included if they met criteria for at least one personality disorder as assessed following the DSM-IV TR (American Psychiatric Association, 1994) criteria and had already attended at least one other evidence-based therapy. Patients were excluded if they were experiencing acute psychotic symptoms, emotional crises which required immediate treatment, severe drug use and untreated substance dependence. Assessments were carried out by Master's level clinical psychology trainees and were recorded so that they could be reviewed (and diagnoses verified) by a doctoral level Clinical Psychologist and accredited schema therapy trainer and supervisor. The Master's level (group) therapists conducted initial intake interviews in order to begin to build rapport with participants with the aim of increasing engagement in the group process. Participants were required to cease all other psychological treatment for the duration of the group. Participants paid a subsidized weekly session fee ($15 AUD). Two participants were diagnosed with BPD, five with avoidant personality disorder and one met criteria for avoidant personality disorder with comorbid schizoid and dependent personality disorders. As can be seen in Table 1, all participants had been treated using a minimum of one evidence-based therapy for at least a year with the average duration of previous treatment being 2.4 years. All participants had a diagnosis of major depressive disorder and seven had clinically significant symptoms of anxiety. No participants were taking anti-depressant medication at any stage of therapy. Table 1 gives basic demographic details of the eight participants and the number of sessions attended.

**Table 1:** **Sociodemographic data**.

	**DSM-IV TR axis II diagnoses**	**MCMI-III clinical syndromes**	**Previous treatment (years)**	**Age**	**Occupation**	**Education level**	**Marital status**	**Sessions attended**
1	Avoidant	Major depression, anxiety	3	28	Student	Honors degree	Single	19
2	Avoidant	Major depression, anxiety	4	47	Unemployed	Secondary school	Single	16
3	Avoidant, schizoid and dependent	Major depression, anxiety	3	37	Unemployed	Secondary school	Single	19
4	Avoidant	Major depression, anxiety	1.5	25	Student	Honors degree	Single	20
5	Avoidant	Major depression, anxiety, somatization	1	29	Unemployed	Diploma	De facto relationship	20
6	Avoidant	Major depression, anxiety	2	42	Unemployed	Year 9, secondary school	Widowed	17
7	Borderline	Major depression, anxiety	3	27	Hospitality	Completing bachelor degree	Single	1
8	Borderline	Major depression	2	35	Health services	Bachelor degree	Relationship	8

MCMI-III, Millon Clinical Multiaxial Inventory; Third Edition (MCMI-III; Millon et al., 2006).

### Treatment

The treatment was adapted from van Vreeswijk and Broersen's (2013) group schema cognitive-behavioral therapy protocol (SCBT-g; van Vreeswijk and Broersen, 2013) but with a significantly greater emphasis on schema mode work and experiential change techniques. The original protocol consisted of eighteen, 90-min weekly sessions with two booster sessions. From the start of the group participants are supported to connect with each other and to create a safe group climate. The first phase of the group focuses on educating clients about the schema model with a focus on their top three schemas and modes. Each week the participants rate the severity of their top three schemas and modes with a focus on reducing the severity of their schemas and modes over the duration of therapy. Schema Mode change strategies are used to increase awareness of schemas and modes, such as identifying mode triggering and challenging the parent modes. Schema mode diaries and flashcards are used to challenge schemas and work on behavioural change within and outside the therapy group. Experiential and interpersonal techniques, such as limited reparenting, chair work and empathic confrontation are also employed. In the second phase of the group, behavioral change techniques, including role plays with healthy adult vs. schemas, cost-benefit analysis, and pie charts are used.

Our adapted model had a strong focus on experiential techniques and mode work for a diagnostically mixed group of personality disorder patients (with a predominant diagnosis of Avoidant personality disorder). ST-g consisted of twenty, 60-min sessions that were run weekly (for 5 months). It was a closed therapy group with provision of five individual sessions during the course of therapy. Sessions were recorded and participants who missed sessions were required to watch the recording before the next session. All sessions were delivered by the same two therapists. Group therapists received one hour of weekly supervision from a doctoral level Clinical Psychologist and accredited schema therapy trainer and supervisor. Consultation was also provided by the fourth author (van Vreeswijk).

The reparenting approach allowed participants to develop a sense of interconnectedness and safety in the initial stages, and in the later stages therapists encouraged participants to develop independence, autonomy, and healthy assertiveness skills in order to meet their emotional needs. Therapists helped participants to develop skills in recognizing their own needs better and to find healthier ways of getting these needs met. In the later stage of treatment, the focus was on motivating and encouraging behavioral change in the present. The “re-familying” component involves the group being encouraged to function as a family where the therapists become a parent figure by reparenting the client, and other group members adopt supportive sibling roles (Farrell and Shaw, 2012). Participants were strongly encouraged to express emotions and ask for the group to meet their emotional needs, rather than coping by detaching from their needs, thoughts and feelings.

We selected specific schema mode focused techniques on the basis of predominant schema modes. Treatment strategies focused on reducing highly avoidant coping mechanisms (i.e., Avoidant/Detached Protector) by using experiential and physical movement exercises to bypass coping. Participants were encouraged to recognize and label the Detached Protector mode when it was active during sessions and when talking about avoidant behavior that had taken place between sessions. To challenge excessively high standards and self-criticism (Demanding Parent), group chair work exercises and group role plays were used. Limited reparenting and “re-familying” exercises were used with child modes (e.g., group imagery). Due to a lack of emotional awareness and emotional tolerance within the group there was an additional focus on increasing awareness, tolerance, and expression of emotions (i.e., Vulnerable Child mode, Healthy Adult mode) e.g., emotion-focused and acceptance exercises. To further increase awareness of schemas and modes and to facilitate emotion regulation, schema-focused mindfulness exercises and regular “mode check-points” (a brief mode awareness exercise) were also incorporated (Kristeller et al., 2006; van Vreeswijk et al., 2014).

Both group therapists had more than 12 months of experience in providing ST to clients with personality disorders and other complex difficulties under the supervision of an accredited schema therapy trainer and supervisor, who regularly observed their psychological therapy skills and checked for treatment fidelity. Both group therapists had attended over 6 days of training in ST.

### Measures

The Millon Clinical Multiaxial Inventory (MCMI-III; Millon et al., 2006) is a self-report questionnaire designed to assess psychopathology, including specific disorders in the DSM-IV. The diagnostic criteria and items used in the MCMI-III inventory parallel that of the DSM-IV (Craig, 2005). Internal consistency of the MCMI-III has been found to be good (0.70–0.80 range), and the test–retest reliability is reported to be good to excellent (around 0.85) (Craig, 2005). Convergent validity and divergent validity have also been found to be good. According to the second edition of the MCMI-III manual (Millon et al., 1997), and using a cut-off of BR = 85, elevated scores on the Personality Scales and Clinical Syndrome Scales provide strong evidence of Axis I and II disorders.

The Young Schema Questionnaire: Short form, second version (YSQ-S2) (Young, 1998) is a 75-item self-report questionnaire that measures 16 core beliefs (early maladaptive schemas). The YSQ-S2 is derived from the original 205-item version (Young and Brown, 1994). Respondents' rate core beliefs regarding oneself or one's relation to others on a six-point scale ranging from 1 (*completely untrue of me*) to 6 (*describes me perfectly*). Research has shown that the YSQ-S2 has good reliability and convergent and discriminant validity (e.g., Schmidt et al., 1995; Rijkeboer et al., 2005). The YSQ-S2 was used to measure change in strength of schemas throughout treatment.

The Schema Mode Inventory (SMI; Young et al., 2007) is a 118-item self-report questionnaire that measures 14 schema modes. Lobbestael et al. (2010) have shown that the SMI has excellent test–retest reliability and satisfactory divergent validity. The internal consistencies of the subscales of the SMI have been found to be good to excellent (*a* ranging from 0.79 to 0.96), and the test–retest reliability is reported to be excellent (Lobbestael et al., 2010). The convergent validity and the divergent validity of the SMI subscales are satisfactory. In the current study we categorized modes into two categories: adaptive schema modes (Healthy Adult and Happy Child) and maladaptive schema modes (all other modes). The SMI was used to measure change in strength of schema modes throughout treatment.

The Symptom Checklist 90-R (SCL-90-R; Derogatis et al., 1973) is a 90-item self-report questionnaire designed to evaluate a broad range of psychological problems and symptoms of psychopathology. The SCL-90-R is widely used to measure participant progress and treatment outcome. Internal consistency of the SCL-90-R has been found to be good to excellent (*a* ranging from 0.77 to 0.96), and the test–retest reliability is reported to be good (Müller et al., 2010). Convergent validity and divergent validity have been found to be good. In this study we have used the SCL-90-R as a measure of general psychological distress.

### Procedure

The YSQ-S2, SMI, and the SCL-90-R were administered at pre-treatment, at mid-treatment (session 10), at end of treatment, and at 6-month follow-up. The MCMI-III was only administered at pre-treatment, at the end of treatment, and at 6-month follow-up. Clinical interview was conducted at pre-therapy and at the end of therapy to assess for personality disorder criteria. To gain an understanding of participants' qualitative experience of participating in ST-g, a focus group was conducted at the end of treatment.

The focus group provided participants with an opportunity to discuss relevant aspects of their experience participating in the ST-g. The focus group was facilitated by the project supervisor. The focus group session was audio and video recorded.

### Statistical analyses

Following visual inspection of the data, a repeated measures analysis of variance (ANOVA) was conducted on all questionnaire measures across the four trial periods (pre, mid, post, and follow-up), and the results of the test are shown in Table 3. To investigate the strength of treatment effects based on all outcome measures, Cohens *d* was calculated. An effect size of 0.2 is considered small, 0.5 is moderate, and 0.8 and above is considered large (Kinnear and Gray, 2008). Due to the single group design, correlation coefficients, and *t*-test statistics for the sample and sample size at each of the time points were considered when calculating the effect sizes. Due to a lack of normative data available on other measures, reliable change and clinical significance was calculated only for the Global Symptom Index (GSI) scale of the SCL-90-R and the SMI. We used the Jacobsen and Truax (1991) approach, where clinical significance was calculated only if reliable change was established. To measure therapy success we used Lambert et al.'s (2008) classification of patients as recovered, improved, unchanged or deteriorated.

## Results

### Attrition

Two participants dropped out of therapy. The first participant dropped out of the group at session three. Feedback provided by this participant suggested that this was due to high levels of shame related to returning to the group after missing a session, and difficulty tolerating the distress associated with working on schemas and maladaptive coping strategies whilst moving house and working full-time. The second participant dropped out at session 16. The participant indicated that this was due to strong feelings of guilt associated with having missed multiple group sessions which triggered a sense of shame and self-criticism for “not putting in enough effort.”

The participants who dropped out reported significantly fewer avoidant personality disorder symptoms at the start of treatment compared with those who completed group therapy: *t*_(6)_ = 4.44, *p* = 0.004 (MCMI-III). The participants who dropped out had a primary diagnosis of BPD and therefore differed diagnostically from the six participants who completed treatment. There were no significant differences between the participants who dropped out and those who remained in group therapy in terms of initial schema and mode severity ratings, depression and anxiety scores and general psychological distress (YSQ-S2: *t*_(6)_ = 0.56, *p* = 0.59; SMI maladaptive modes: *t*_(6)_ = 0.09, *p* = 0.93; SMI adaptive modes: *t*_(6)_ = 0.23, *p* = 0.51; depression: *t*_(6)_ = 1.27, *p* = 0.25; anxiety: *t*_(6)_ = 1.71, *p* = 0.14; general psychological distress: *t*_(6)_ = 1.51, *p* = 0.24). The participants who dropped out were excluded from the analyses and the analyses were carried out on treatment completers only.

### Visual inspection

**Figure 1:**
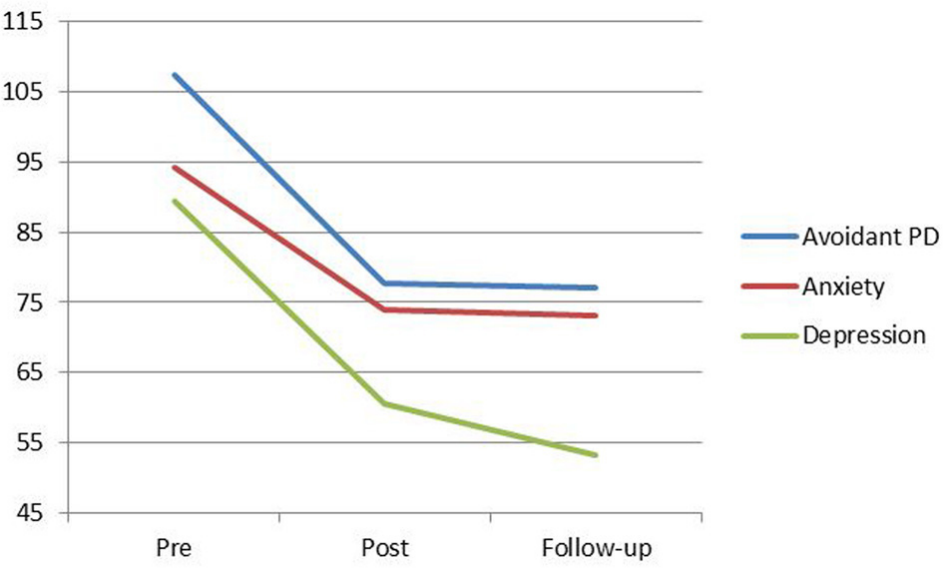
**MCMI-III Avoidant PD, Anxiety, and Depression means from pre to post-treatment, and follow-up**. Scores above 85 indicate the persistent presence of personality disorder indicators.

Visual inspection of the data reveals that at the end of treatment, four clients no longer met criteria for avoidant personality disorder based on scores on the MCMI-III (Millon et al., 2006) (Figure 1). These treatment gains were maintained at 6-month follow-up. At 6-month follow-up a fifth group member no longer met criteria for avoidant personality disorder. These findings are consistent with client self reports of improved daily functioning and reduced avoidant coping behaviors. Pre-post scores also show a large reduction in depression and anxiety levels. The six clients who completed treatment no longer met criteria for depression at post-treatment. These treatment gains were not only maintained at 6-month follow-up but continued to decline. For three clients, anxiety symptoms reduced to the non-clinical range.

**Figure 2:**
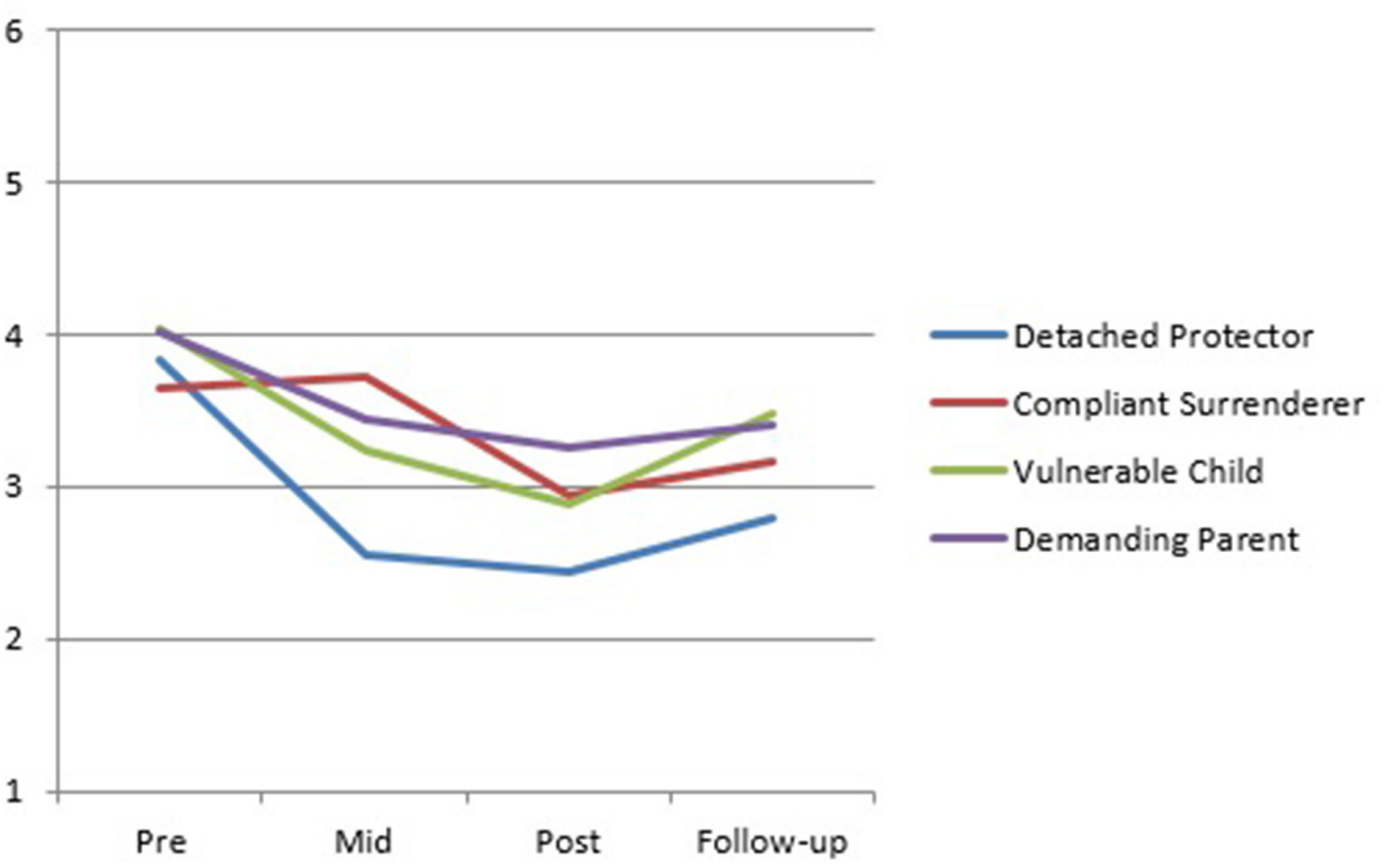
**SMI maladaptive modes group means from pre-treatment through mid-, post-, and follow-up**.

**Figure 3:**
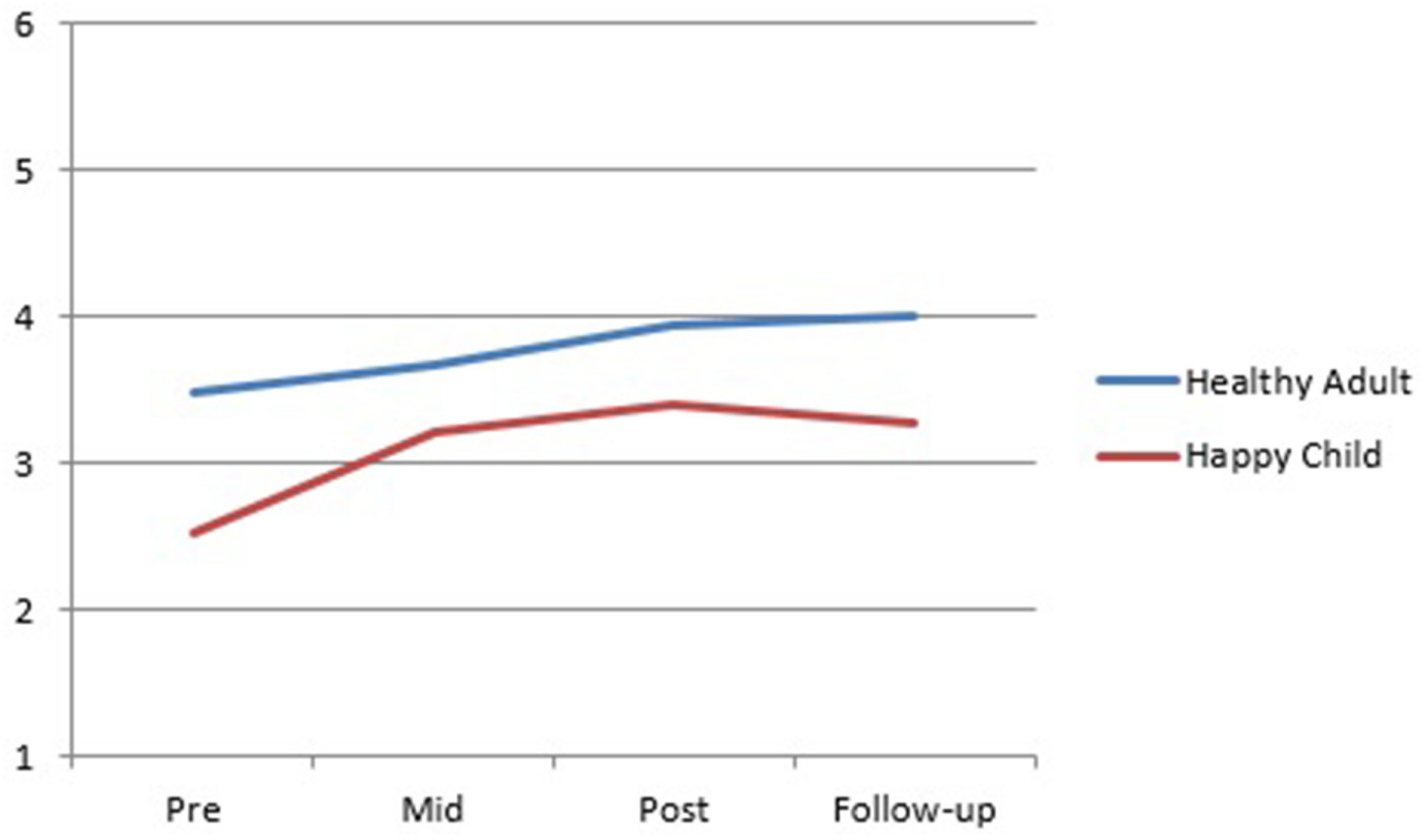
**SMI adaptive modes group means from pre-treatment through mid-, post-, and follow-up**.

**Figure 4:**
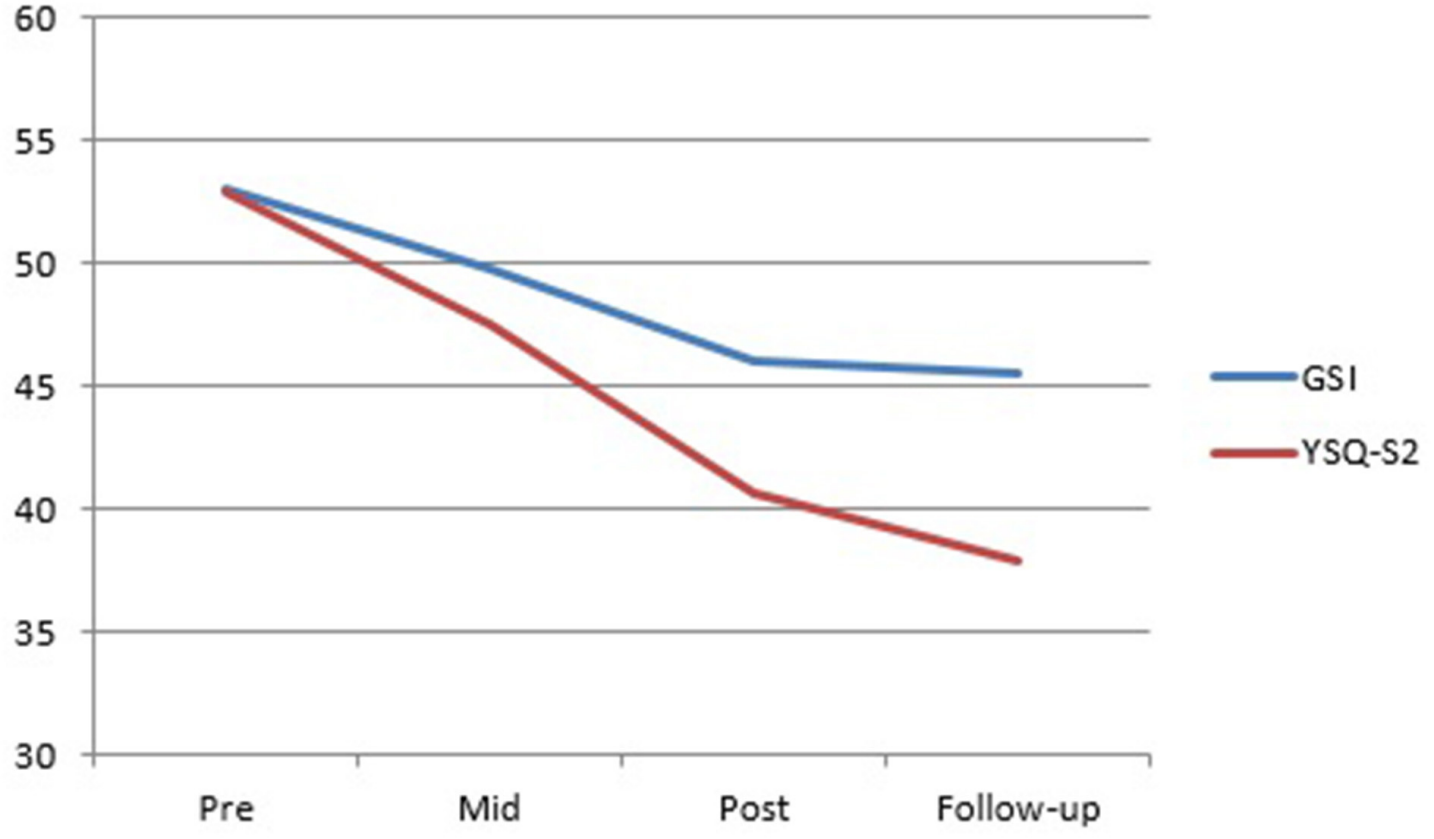
**Total YSQ-S2 and GSI group means from pre-treatment through mid-, post-, and follow-up**.

Visual inspection of the SMI group mean scores indicates a trend for the Detached Protector, Compliant Surrenderer and Vulnerable Child to reduce across treatment with a slight increase in the Vulnerable Child from post to follow-up (Figure 2). The Happy Child and Healthy Adult increased across treatment with a slight decrease in the Happy Child mode from post to follow-up (Figure 3). Total YSQ-S2 scores show improvement across treatment phases (Figure 4). Group scores on the Global Severity Index (GSI) of the SCL-90-R showed a downward trend across all treatment phases (Figure 4).

### Cross-sectional associations among study variables

First we determined Pearson correlations among scores of the GSI, YSQ-S2 (total score), SMI adaptive modes and SMI maladaptive modes before therapy. As can be seen in Table 2, there was a significant association between symptomatic distress as assessed by the GSI of the SCL-90-R and YSQ-S2 at baseline (*r* = 0.82, *p* < 0.05). Moreover, adaptive schema modes were negatively related to YSQ-S2 total scores (*r* = −0.90, *p* < 0.05). There was also a significant association between avoidant scores and maladaptive schema modes (*r* = 0.90, *p* < 0.05). The other study variables did not correlate with each other.

**Table 2:** **Pearson correlations between GSI scores, depression, anxiety, avoidant, YSQ-S2, and SMI at baseline**.

**Measure**	**1**	**2**	**3**	**4**	**5**	**6**	**7**
1. GSI							
2. YSQ-S2	0.60						
3. SMI maladaptive	0.82^*^	0.69					
4. SMI adaptive	−0.68	−0.90^*^	−0.7				
5. Depression	−0.06	0.31	0.08	0.13			
6. Anxiety	−0.13	−0.60	−0.5	0.27	−0.81		
7. Avoidant	0.77	0.63	0.90^*^	−0.63	0.22	−0.60	

The diagonal shows correlation coefficients; N = 6; GSI, global symptom index of the SCL-90-R; YSQ-S2, YSQ-S2 total score; SMI Maladaptive, Schema Mode Inventory total score of maladaptive schema modes, SMI Adaptive, Schema Mode Inventory total score of adaptive schema modes;

* Correlation significant at p < 0.05 (two-tailed).

**Table 3:** **Means, standard deviations, F statistics and *p*-value's of all questionnaire measures as a result of the repeated measures ANOVA**.

**Measure**	**Pre (SD)**	**Mid (SD)**	**Post (SD)**	**Follow-up (SD)**	***F* (*p*-value)**
GSI	53 (6.54)	49.83 (5.71)	46 (6.72)	45.50 (6.57)	6.37, *p* = 0.03^*^
YSQ-S2	52.87 (5.84)	47.5 (6.37)	40.63 (5.26)	37.90 (5.23)	27.43, *p* = 0.001^**^
SMI maladaptive	36.68 (4.15)	33.08 (1.89)	30.46 (3.15)	30.74 (2.87)	8.49, *p* = 0.02^*^
SMI adaptive	6.02 (0.91)	6.88 (1.23)	7.35 (1.10)	7.27 (1.12)	3.56, *p* = 0.10
Depression	89.50 (3.56)		60.5 (13.63)	53.33 (20.67)	19.86, *p* = 0.002^*^
Anxiety	94.17 (6.62)		74 (36.89)	73 (36.11)	2.23, *p* = 0.19
Avoidant	107.5 (8.43)		77.67 (11.52)	77 (11.22)	36.33, *p* < 0.001^**^

* Indicates significance to the 0.05 level;

** Indicates significance to the 0.01 level.

As the study aimed to investigate change in the maladaptive schemas of the participants across treatment phases, the results of the repeated measures ANOVA for the YSQ-S2 indicated a significant time effect: *F*_(1.36,6.82)_ = 27.43; *p* = 0.001. Therefore, on the basis of the repeated measures ANOVA, the findings suggest that the group's scores as a whole significantly improved for five of seven of the questionnaire measures (the YSQ-S2, the MCMI-III avoidant subscale, the MCMI-III depression subscale, the SMI maladaptive modes and the GSI of the SCL-90-R). The group scores of the Anxiety component of the MCMI-III showed a positive trend, though not statistically significant. The group scores of the SMI adaptive modes showed a trend for the SMI adaptive modes to increase across treatment, with a slight decrease in the SMI adaptive modes from post to follow-up.

Effect size (*d*) calculations in all questionnaire measures are shown in Table 4. Effect sizes between pre-therapy and post-therapy, and pre-therapy and follow-up were all large. Effect sizes from post to follow-up were small to moderate.

**Table 4:** **Effect sizes (*d*) in all questionnaire measures, showing both pre to post effects and pre to follow-up effects**.

**Treatment period**	**YSQ-S2 (*d*)**	**Avoidant (*d*)**	**Anxiety (*d*)**	**Depression (*d*)**	**GSI (*d*)**	**SMI adaptive (*d*)**	**SMI maladaptive (*d*)**
Pre to post	2.20	2.96	0.76	2.91	1.06	1.32	1.69
Pre to follow-up	2.70	3.07	0.82	2.44	1.14	1.22	1.66
Post to follow-up	0.52	0.06	0.03	0.41	0.08	(0.07)	(0.09)

Use of brackets () denotes that the change demonstrated moved in the opposite direction from what would be clinically desirable (i.e., the group mean score worsened over time). Guideline cut-off scores for the magnitude of effect size: 0.2–0.5 = small; 0.5–0.8 = medium; 0.8 and above = large.

Reliable change and clinical significance were calculated for the SMI and the GSI of the SCL-90-R. For the SMI adaptive modes, from pre-therapy to post-therapy, two (33.33%) participants recovered, one (16.67%) improved, and three (50%) remained the same. For the SMI maladaptive modes, from pre-therapy to post-therapy, one (16.67%) participant recovered, and five (83.33%) improved. For the GSI, from pre-therapy to post-therapy, two (33.33%) participants recovered and four (66.67%) improved. By follow-up, three (50%) participants recovered, two (33.33%) improved and one (16.67%) remained unchanged (one participant improved from pre-therapy to post-therapy, but due to an increase in scores from post-therapy to follow-up was considered unchanged overall from pre-therapy to follow-up).

### Qualitative analysis

The video recorded data (of the focus group) was viewed a number of times and themes were identified and agreed upon by all authors. Themes were selected on the basis of frequency. Illustrative verbatim quotes are shown with the relevant themes below. Data analysis revealed four overarching themes: (1) the normalizing effect of ST-g, (2) ST-g actively challenges schemas, (3) disinhibition effect of ST-g, and (4) motivational influence of ST-g.

### The normalizing effect of ST-g

Nearly all participants reported that ST-g normalized their schemas and associated emotional experiences. For instance:

“Everyone has schemas.”
“When I see that I can be compassionate toward others who have schemas, I then start to challenge my own.”

### ST-g actively challenges schemas

Participants explained that ST-g increases understanding of self through observation of others and allows direct experience in practicing challenging schemas just by attending. Some verbatim quotes that illustrate this are the following:

One participant with the social isolation schema reported “When I noticed others sharing and expressing themselves, it helped me feel safe enough to participate. Seeing others with similar schemas helps me feel I am similar to others.”
Another participant with an entitlement schema reported “I notice others' needs more.”
A participant with the self-sacrifice schema said “I began to think about what I need from other group members.”

### Disinhibition effect of ST-g

Another theme frequently endorsed by participants was that the group actually encourages and evokes self-expression. For example:

“Seeing others being vulnerable and not judging each other, allowed me to feel safe to express my feelings and needs.”

### Motivational influence of ST-g

A majority of participants noted that ST-g motivated them to make behavioral changes. For instance:

“Seeing others make progress has more impact on me (compared to individual therapy)”
“others' progress spurs me on.”
“There is a lot of emphasis on the steps needed to make (behavioral) changes and build my healthy self.”

## Discussion

This study aimed to investigate the feasibility, acceptability and preliminary efficacy of ST-g in a sample of eight participants with mixed personality disorders and comorbidity. A large effect size was found for the group between pre-therapy and follow-up for all measures. All outcome measures showed changes with large effect sizes in avoidant personality disorder symptom severity, and depression and anxiety levels between pre-therapy and follow-up. Four participants achieved a loss of personality disorder diagnosis at the end of therapy. By follow-up, five participants had achieved a loss of diagnosis, suggesting that participants derived ongoing benefits from the group even after treatment ended. Six participants no longer met criteria for depression at the end of treatment and this was maintained for all participants at 6-month follow-up. Clinically significant change on the GSI at follow-up showed that a majority of participants had recovered. For the SMI maladaptive modes, the majority of participants showed improvement at follow-up. Clinically significant change for the SMI adaptive modes at follow-up showed that 40% of the participants had recovered.

One of the main aims of ST is to help patients to reduce maladaptive coping modes so that they can begin to find healthy ways of enabling their emotional needs to be met, both by others and themselves (Young et al., 2003). In this study, the largest change pre-post was in the Detached Protector mode, suggesting a reduction in numbing or distancing as a way of dealing with emotions and relationships. As patients notice an initial increase in awareness of overwhelming emotions, they often initially fall back on old familiar coping modes as a way of managing. It is a common clinical observation that participants' scores on the Vulnerable Child mode (i.e., the state in which they are in touch with emotions) often increase across the first-half of therapy as clients become less emotionally detached and gain increased awareness of their early maladaptive schemas and schema modes. It seems possible that pre-treatment scores on the SMI may not in fact reflect actual mode severity (i.e., under rating). Interestingly, participants rated themselves with relatively high levels of “Healthy Adult” mode at pre-treatment, in spite of significant pathology and longstanding psychological difficulties. Clinical observations in the group suggested that this may well have indicated an initial confusion by group participants over the differences between healthy and avoidant coping, resulting in a tendency to overestimate their Healthy Adult mode. Apparent limited change in maladaptive schema modes may also indicate increased awareness of emotional states across therapy. The significant reduction in DSM-IV-TR avoidant personality disorder criteria may reflect behavioral change, whereas longer treatment may be required to achieve change at an emotional level associated with schema modes. It may be that more than 20 sessions is required to activate lasting emotional change in this population. Future studies should increase the number of sessions and duration of sessions in order to allow more scope for experiential work to take place in the group context.

In this study two participants dropped out of therapy. Comparison with attrition rates in other group studies suggests that this is may be a relatively low attrition rate and is comparable with similar studies using ST (Davis et al., 2006; Simpson et al., 2010; van Vreeswijk et al., 2012). The two participants who dropped out in this study had a primary diagnosis of BPD (and fewer symptoms of avoidant personality disorder than other participants) and therefore differed diagnostically from the six participants who completed treatment. It seems plausible that when these participants expressed emotions openly in early sessions, at a time when the rest of the participants were detached from their feelings, this may have triggered social isolation and defectiveness schemas which may in turn have been experienced as alienating for these two participants. This finding may have important clinical implications regarding the potential value of ensuring that participants are screened carefully to ensure that all share schema mode constellations with at least half of the group, in order to prevent re-experiencing of old patterns in relation to feeling excluded or different in some way from others. In addition, addressing shame explicitly may be important in helping clients to manage feelings of shame that may otherwise contribute to early drop-out. Lastly, encouraging clients to connect with other group members should be a priority in order to prevent re-experiencing of old patterns related to feeling interpersonally disconnected or isolated.

The clinical improvements demonstrated in this study indicate that ST delivered in a group setting may hold promise for participants with different personality disorders and high levels of comorbidity. It has been hypothesized that there may be specific factors operating in a group setting that challenge schemas at a group-process level (Simpson et al., 2010). The qualitative feedback collected in this study, indicates that the group helps to normalize participants' psychological experiences and difficulties and promotes self-expression and self-disclosure, which, in turn, reduces inhibition (i.e., bypasses the “Detached Protector”). The group fostered the development of psychological insight and increased self-awareness as participants observed their own schemas in other participants' and the way those schemas influence interaction between group members. The unique social component of the group directly challenges a number of participants' schemas by facilitating social interaction and helping to build new healthier models of social relationships that challenge participants' existing life patterns.

The group also motivated participants to make behavioral changes that they had been unable to make during individual therapy. Participants felt motivated by directly observing other participants' progress. Some of the themes identified through the focus group have been previously identified in the literature (e.g., Farrell et al., 2009; Simpson et al., 2010), such as the normalizing and de-shaming aspect of ST-g. These potential additive effects of group therapy could be explored further by comparing group with individual schema therapy.

Limitations of this study include a small sample size and a lack of a longer follow-up period. Moreover, the fact that a mixed personality disorder group was used reduced our number of participants further. If our study had focused on a single diagnostic group, this may have allowed greater standardization of assessments and facilitated the use of specific cut-off points for inclusion in the study, thereby improving the methodological robustness of the study. However, this may also have reduced the generalizability of this study to general clinical populations seen within health and hospital-based clinics that are often composed of heterogeneous client groups. In addition, due to the fact that this was a small pilot study that was conducted to determine the feasibility and efficacy of short-term ST-g with a mixed personality disorder population, a comparative control group was not included. As such, it is not possible to determine from these findings the extent to which the results are due to participants' response to therapy in general or to ST-g specifically or to other variables. Although DSM-IV criteria were utilized when interviewing and diagnosing psychiatric disorders, a formal diagnostic interview such as the SCID-I and II would have been a more robust means of assessment at pre-treatment. Additionally, in this study, we did not set a predetermined MCMI-III cut off point for inclusion. Finally, although treatment fidelity was rated by accredited schema therapists regularly throughout the study, no formal ST-adherence rating was done. Future studies in this area could improve on our procedure by assigning random tapes to raters not involved in the study. If it was a large trial, we would complete intention-to-treat analyses by including participants who dropped-out in all analyses.

This naturalistic pilot study allows greater exploration of the level of change possible over a 20 week schema therapy group with a mixed personality disorder sample. Naturalistic designs are clinically useful and have high ecological validity, which can allow results to be generalized to patients generally seen in typical clinical settings (i.e., community mental health teams, hospital wards) (Lincoln and Guba, 1985; van Vreeswijk et al., 2012). In addition, this study contributes valuable data which will inform the development of a larger RCT with this population. In particular, this study may have implications for the significant number of patients with personality disorders who may not respond to conventional treatments offered within many health service settings. This study contributes to a growing body of literature that suggests that ST-g shows promise as an intervention which may stimulate avoidant coping patterns through experiential, cognitive and behavioral group processes, many of which appear to be unique to working in a group setting.

### Conflict of interest statement

The authors declare that the research was conducted in the absence of any commercial or financial relationships that could be construed as a potential conflict of interest.
